# Inherited myogenic abilities in muscle precursor cells defined by the mitochondrial complex I-encoding protein

**DOI:** 10.1038/s41419-023-06192-2

**Published:** 2023-10-19

**Authors:** Norio Motohashi, Katsura Minegishi, Yoshitsugu Aoki

**Affiliations:** Department of Molecular Therapy, National Institute of Neuroscience, National Center of Neurology and Psychiatry (NCNP), Tokyo, 187-8502 Japan

**Keywords:** Senescence, Stem-cell research, Skeletal muscle, Muscle stem cells, Energy metabolism

## Abstract

Skeletal muscle comprises different muscle fibers, including slow- and fast-type muscles, and satellite cells (SCs), which exist in individual muscle fibers and possess different myogenic properties. Previously, we reported that myoblasts (MBs) from slow-type enriched soleus (SOL) had a high potential to self-renew compared with cells derived from fast-type enriched tibialis anterior (TA). However, whether the functionality of myogenic cells in adult muscles is attributed to the muscle fiber in which they reside and whether the characteristics of myogenic cells derived from slow- and fast-type fibers can be distinguished at the genetic level remain unknown. Global gene expression analysis revealed that the myogenic potential of MBs was independent of the muscle fiber type they reside in but dependent on the region of muscles they are derived from. Thus, in this study, proteomic analysis was conducted to clarify the molecular differences between MBs derived from TA and SOL. NADH dehydrogenase (ubiquinone) iron-sulfur protein 8 (Ndufs8), a subunit of NADH dehydrogenase in mitochondrial complex I, significantly increased in SOL-derived MBs compared with that in TA-derived cells. Moreover, the expression level of *Ndufs8* in MBs significantly decreased with age. Gain- and loss-of-function experiments revealed that *Ndufs8* expression in MBs promoted differentiation, self-renewal, and apoptosis resistance. In particular, *Ndufs8* suppression in MBs increased p53 acetylation, followed by a decline in NAD/NADH ratio. Nicotinamide mononucleotide treatment, which restores the intracellular NAD^+^ level, could decrease p53 acetylation and increase myogenic cell self-renewal ability in vivo. These results suggested that the functional differences in MBs derived from SOL and TA governed by the mitochondrial complex I-encoding gene reflect the magnitude of the decline in SC number observed with aging, indicating that the replenishment of NAD^+^ is a possible approach for improving impaired cellular functions caused by aging or diseases.

## Introduction

Skeletal muscle shows great potential to regenerate after severe damage caused by sports training or diseases. Muscle satellite cells (SCs), which are mononuclear myogenic stem cells, are critical factors determining muscle regeneration [1, 2]. Maintaining the number and ability of muscle SCs is essential to ensure continued homeostasis and achieve muscle hypertrophy or regeneration. Although SC ablation did not affect sarcopenia, these cells are necessary for exercise-induced muscle hypertrophy [3, 4], maintenance of neuromuscular junctions [5], or muscle regeneration [6, 7]. However, the number of SCs and their proliferative potential significantly decline because of aging [8, 9] and muscle diseases, such as Duchenne muscular dystrophy (DMD) [10]. The decline in the number of SCs might be due to the impairment of myogenic cell abilities with age or diseases [8, 11–14], and the deterioration of environmental conditions or niche with surrounding SCs might affect the number of SCs [15, 16]. Previous studies using mice and human samples demonstrated that the age-related reduction of muscle SC content was more severe in fast-type muscle fibers than in slow ones [9, 17]. In addition, SCs in slow-type muscle fibers showed great potential to maintain their number independent of their niche [18]. These observations supported the hypothesis that SCs from slow-type muscle fibers could maintain their number. However, whether myogenic abilities in SCs are determined by fiber types or the region they reside remains unclear as muscle fiber types are transitioned frequently because of several situations, including denervation. Some studies using denervation muscles demonstrated the alteration of muscle fiber type distribution [19], whereas some showed no significant changes even after muscle injury [20, 21]. Therefore, the relationship between SCs and fiber types remains unknown.

Previously, we reported that myoblasts (MBs) derived from slow-type enriched soleus (SOL) generated slow-type myotubes, whereas MBs derived from fast-type enriched tibialis anterior (TA) formed fast-type myotubes in vitro and in vivo [18]. In this study, considering the relatively high proportion of type IIb fibers in TA and the enrichment of type I fibers in SOL, we selected TA and SOL to represent fast-type fibers and slow-type fibers, respectively [18]. Furthermore, we observed a high expression level of Tbx1 in TA-derived MBs compared with that in SOL-derived MBs, and Tbx1 played a role in modulating muscle fiber types and oxidative metabolism in myotubes [18]. However, Tbx1 expression did not explain the differences in self-renewal potential between SOL and TA MBs [18]. Thus, accumulating studies have been published to elucidate the SC self-renewal mechanism by targeting several factors, including growth factors or niches surrounding SCs [16]. However, previous experiments have revealed that the self-renewal ability of SOL and TA MBs was not dependent on recipient muscles, and that the intrinsic potential of SCs could govern the maintenance of their abilities. Several factors, including p38 or Stat3, have been found to play a role in SC self-renewal, and the expression of such factors was independent of the niche [11, 12, 22, 23].

In this study, we have clarified that SCs were isolated from denervated muscles, and their inherited properties in SCs were independent of neural adaptation. Using proteomic analysis, this study identified that a mitochondrial complex I-encoding protein, NADH dehydrogenase (ubiquinone) iron-sulfur protein 8 (Ndufs8), significantly increased in SOL MBs compared with that in TA MBs, indicating its high potential to self-renew. Gain- and loss-of-function experiments revealed that Ndufs8 expression modulated myogenic differentiation, apoptosis, and metabolism in MBs mediated by the modulation of the Sirtuin (Sirt)-p53 signaling cascade depending on the NAD/NADH ratio, which is regulated by Ndufs8. Further, supplementation of NAD^+^ could accelerate self-renewal potential in SCs. These observations highlighted the functional differences between SOL and TA-derived MBs mediated by Ndufs8 and indicated that NAD^+^ supplementation is a potential approach for maintaining the SC pool.

## Results

### Myogenic cell properties are governed by the muscle fiber region in which they reside, independent of the muscle fiber shift by nerve stimulation

**Fig. 1:**
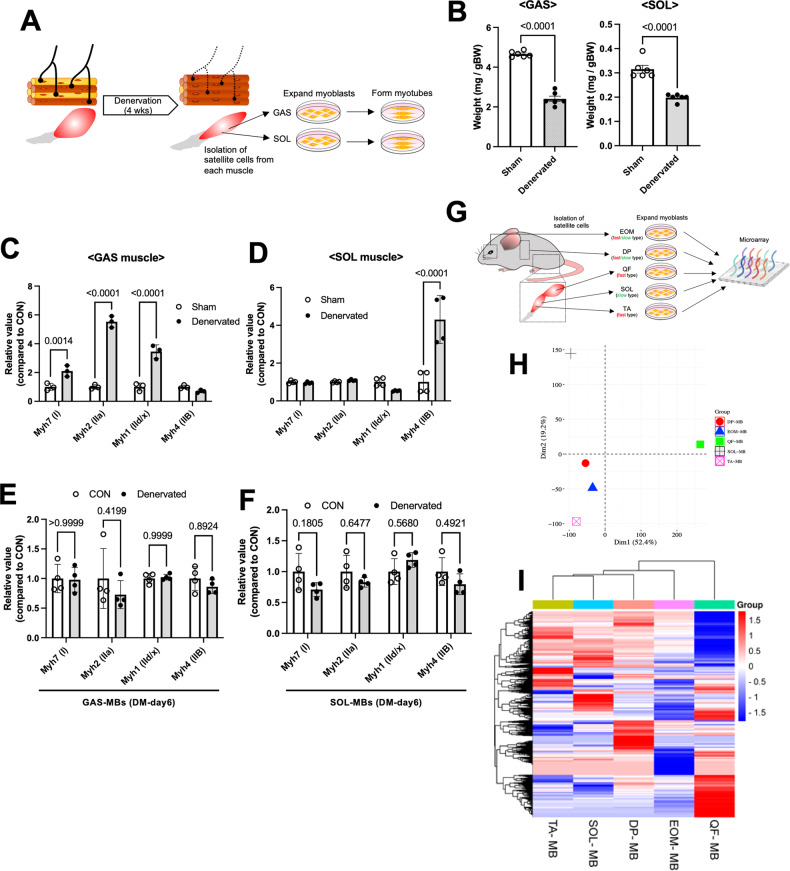
Denervation does not affect the myogenic properties of MBs from fast- and slow-type muscles. **A** Scheme of cell differentiation experiments using SCs from denervated muscles. **B** The muscle weight of GAS and SOL was quantified. Data are presented as the mean ± SE (*n* = 6). **C** and **D** The expression level of *Myh7*, *Myh2*, *Myh1*, and *Myh4* in GAS (**C**) and SOL (**D**) was quantified. The expression values were normalized to *GAPDH* expression. Data are presented as the mean ± SE (*n* = 4). **E** and **F** The fiber type distribution of myotubes from denervated GAS (**E**) and SOL (**F**) was quantified by qPCR. Data are presented as the mean ± SE (*n* = 4). **G** Scheme of gene expression analysis of SCs obtained from normal EOM, DP, QF, TA, and SOL. **H** and **I** PCA plot (**H**) and heatmap (**I**) were generated from gene expression analysis using MBs derived from EOM, DP, QF, TA, and SOL of normal muscles.

Based on previous reports, the potential of myogenic cells from each muscle fiber type varies regardless of the environmental condition or niche surrounding SCs [18], whereas whether changes in the muscle fiber type induced by several circumstances, including nerve stimulation, can govern the ability of muscle SCs remains unknown. Thus, in this study, SCs were collected from denervated fast-type gastrocnemius (GAS) and SOL in C57/BL6 wild-type (WT) mice (12 weeks old) and cultured under growth conditions in vitro (Fig. 1A). Four weeks after denervation, muscle weight in denervated GAS and SOL significantly decreased compared with sham-operated control muscles (Fig. 1B), and the distribution of muscle fiber types dramatically shifted from fast to slow-type in GAS (Fig. 1C) or from slow to fast-type in SOL (Fig. 1D). After SC expansion from denervated GAS and SOL, cultured MBs were induced for differentiation for 6 days (DM day 6), and the expression of myosin heavy chain (MyHC) isoforms (I, IIa, IId/x, and IIB) was examined by quantitative polymerase chain reaction (qPCR). No significant changes in muscle fiber type distribution in myotubes from GAS- or SOL-derived MBs were observed (Fig. 1E and F), although fiber types in denervated muscle shifted after 4-week denervation (Fig. 1C and D). Based on previous results, that is, myogenic cells from each muscle fiber were predetermined to undergo differentiation into a specific muscle fiber type [18], these observations strongly supported the hypothesis that the abilities of myogenic cells derived from individual fiber types maintained their different potential regardless of nerve stimulation.

This study also investigated whether the abilities of SCs or MBs depend on muscle fiber types where SCs reside or on the region of the muscle where SCs are derived. In clarifying this hypothesis, this study performed gene expression analysis of MBs from fast-type quadriceps femoris (QF), TA, slow-type SOL, and fast/slow-type extraocular (EOM), and diaphragm muscles (Fig. 1G). The principal component analysis (PCA) data and heatmap generated from microarray analysis demonstrate that the gene expression patterns differ depending on the origin of MBs (Fig. 1H and I).

### Distinct properties of myogenic cells from SOL and tibialis muscles

**Fig. 2:**
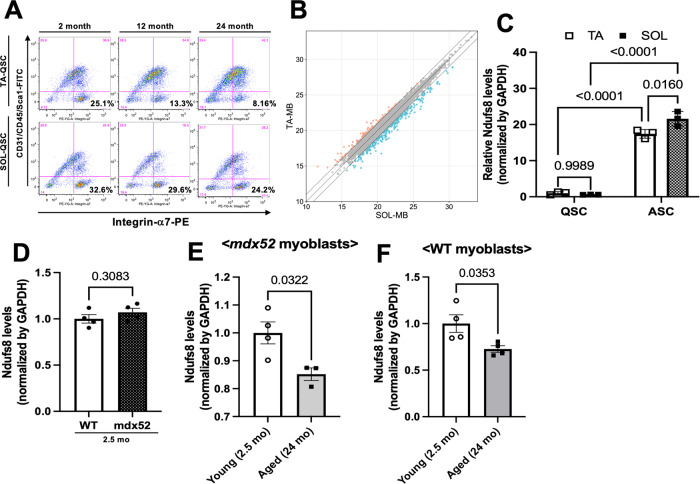
Proteomic analysis reveals myogenic cell diversity in TA- and SOL-derived cells. **A** Freshly isolated mononuclear cells derived from TA and SOL of different ages (2, 12, and 24 months) were stained against CD31/CD45-FITC and integrin-α7-APC. CD31/CD45(−) and integrin-α7(+) cells were defined as QSCs. **B** Differentially expressed proteins in MBs derived from TA and SOL were visualized by scatter plots. **C**–**E** The expression levels of *Ndufs8* in QSCs and ASCs from TA and SOL (**C**), in MBs from WT and *mdx52* limb muscles (**D**), and in *mdx52* MBs from young (2.5 months) and aged (24 months; **E**) limb muscles were quantified by qPCR. **F** The expression levels of *Ndufs8* in WT MBs from young (2.5 months) and aged (24 months) limb muscles were quantified by qPCR. Data are presented as the mean ± SE (*n* = 3–4).

Based on previous reports, the number of SCs declined with age, whereas the age-related reduction of muscle SC content was more prominent in fast-type muscle fibers than in slow ones [9, 17]. In confirming whether the number of SCs in fast-type TA and slow-type SOL reduces with age (from 2 to 24 months old), mononuclear cells were collected from fast-type TA and slow-type SOL individually, and the number of integrin-α7(+) quiescent SCs (QSCs) was analyzed by flow cytometry. As shown in Fig. 2A, the number of QSCs from TA and SOL decreased with age, but the degree of reduction was less in those from SOL (Fig. 2A), which is consistent with previous results [9, 17].

Proteomic profiling was assessed by data-independent acquisition (DIA) mass spectrometry in MBs from TA and SOL to clarify the molecular differences among them. We could identify several proteins that were increased and decreased in SOL MBs compared with TA MBs (Fig. 2B). In particular, Gene Ontology (GO) biological component analysis demonstrated that SOL MBs are enriched with proteins related to mitochondrial respiratory complex I, and GO molecular function analysis indicated that the highly expressed proteins in SOL MBs are involved in NADH dehydrogenase activity (Supplementary Fig. S1A and B). Thus, this study selected Ndufs8, a subunit of NADH dehydrogenase, as a possible protein to define the functional differences in MBs from TA and SOL.

First, in quantifying *Ndufs8* expression in QSCs and activated SCs, SCs were collected from TA and SOL, and *Ndufs8* expression was assessed. *Ndufs8* expression in QSCs from TA and SOL was quite low, but it significantly increased in activated SCs from TA and SOL and was significantly higher in SOL-derived cells than in TA MBs (Fig. 2C). In addition, *Ndufs8* expression was evaluated using MBs from DMD model *mdx52* mouse muscle, whose SC number declined with age [10]. No significant differences in MBs from WT and *mdx52* muscles were observed (Fig. 2D), indicating that dystrophin deficiency did not affect *Ndufs8* expression. However, this expression in MBs from aged (24 months old) *mdx52* muscles significantly declined compared with those from young (1.5 month old) *mdx52* muscles (Fig. 2E). We isolated MBs from the muscles of young (2.5 months old) and aged (24 months old) WT mice to investigate the impact of sarcopenia on Ndufs8 expression in MBs. Subsequently, we analyzed the expression of Ndufs8 and observed a significant decrease in Ndufs8 expression in aged MBs compared with their young ones (Fig. 2F). These observations indicated that *Ndufs8* expression reflects the biological differences in myogenic cells derived from TA and SOL, and it is possibly affected by sarcopenia.

### Ndufs8 expression regulates myogenic properties in MBs

**Fig. 3:**
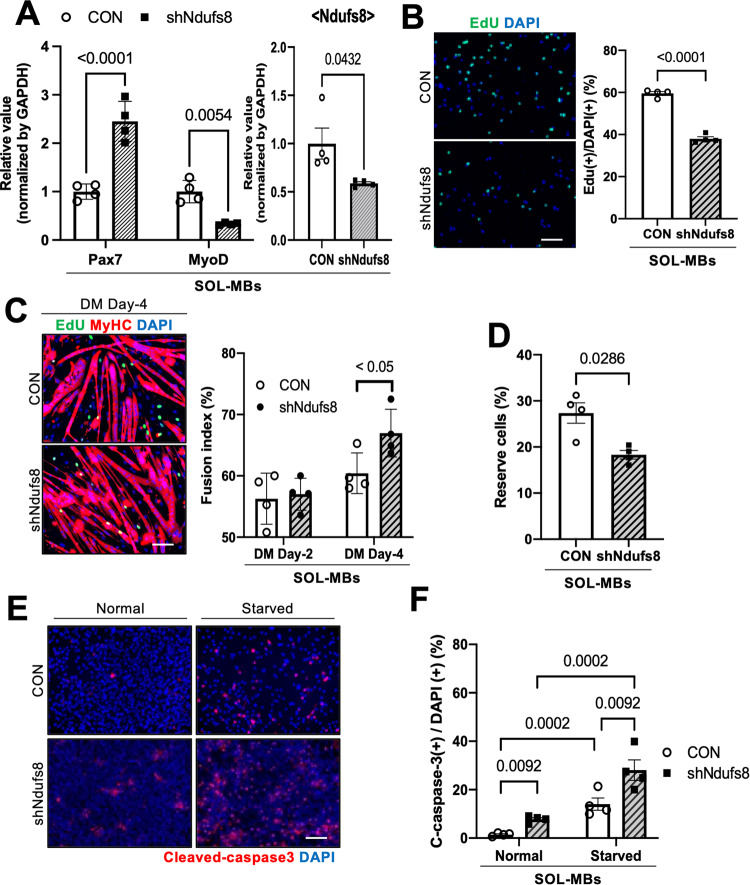
Ndufs8 inhibition impairs myogenic abilities and apoptosis resistance. **A** *Pax7*, *MyoD*, and *Ndufs8* expression in sh*Ndufs8*-infected MBs from SOL as quantified. Data are presented as the mean ± SE (*n* = 4). **B** *Ndufs8*-suppressed MBs were cultured in a growth medium with EdU. The number of EdU(+) cells was counted. Data are presented as the mean ± SE (*n* = 4). Scale bar, 100 µm. **C** and **D** *Ndufs8*-suppressed MBs were cultured in a differentiation medium for 4 days and stained against EdU (green) and MyHC (red) with DAPI (blue). The proportion of MyHC(+) cells among total nuclei (**C**) and the number of MyHC(−)EdU(−) reserve cells (**D**) were quantified. Data are presented as the mean ± SE (*n* = 4). Scale bar, 100 µm. **E** *Ndufs8*-inhibited MBs were cultured under normal or serum-starved conditions for 12 h and stained against cleaved caspase-3 (red) with DAPI (blue). **F** The proportion of cleaved caspase-3(+) cells among total nuclei were quantified. Data are presented as the mean ± SE (*n* = 4). Scale bar, 100 µm.

Next, *Ndufs8* expression was suppressed by a sh*Ndufs8* lentivirus vector in SOL-derived MBs to evaluate the effect of *Ndufs8* expression on the function of myogenic cells (Fig. 3A). qPCR experiments revealed that myogenesis-related *MyoD* expression decreased, whereas *Pax7* expression increased in *Ndufs8*-suppressed MBs compared with control (Fig. 3A). A proliferation assay experiment showed that the number of EdU(+)-proliferating cells significantly decreased in sh*Ndufs8-*treated MBs compared with control (Fig. 3B). Four days after the induction of MB differentiation, the differentiation index was calculated by determining the percentage of DAPI(+) nuclei within MyHC-positive myotubes relative to the total number of DAPI(+) nuclei. We found that the number of MyHC(+) myofibers significantly increased (Fig. 3C), whereas the number of MyHC(−)EdU(−) reserve cells, which are equivalent to self-renewing SCs in vitro [24–27], significantly decreased in *Ndufs8*-inhibited MBs compared with control (Fig. 3D). In addition, the number of apoptotic cells was evaluated by staining with a cleaved caspase-3 antibody in MBs under normal and serum-starved conditions. As shown in Fig. 3E and F, the number of apoptotic cells in *Ndufs8*-inhibited MBs significantly increased compared with control (Fig. 3E and F). These results suggested that *Ndufs8* inhibition increases myogenic differentiation and decreases the capacity of reserve cell formation and apoptosis resistance.

**Fig. 4:**
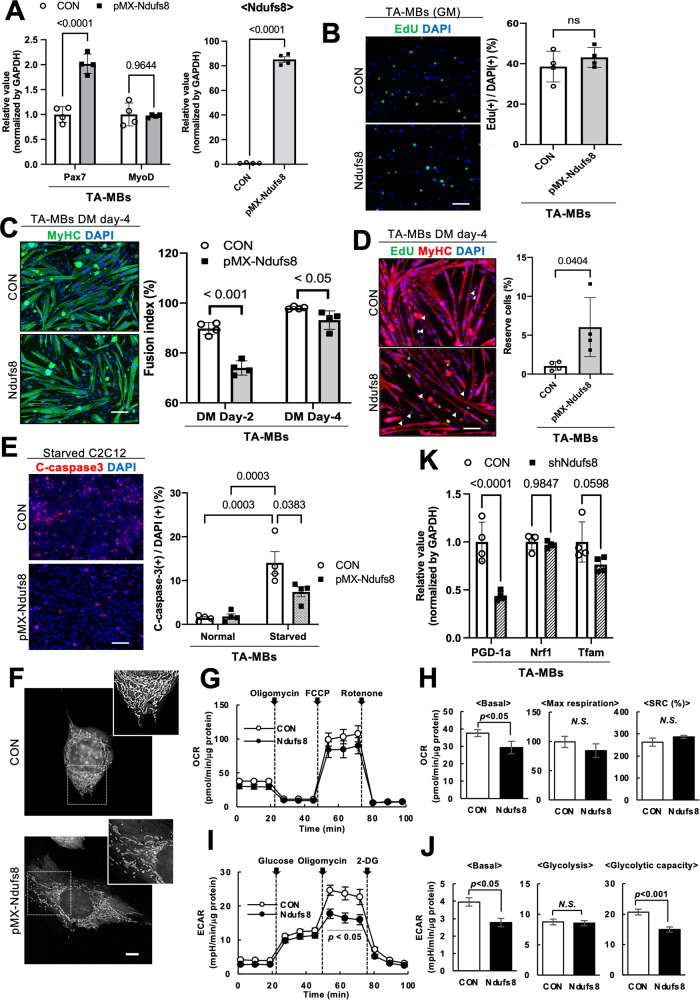
Ndufs8 overexpression stimulates myogenic abilities and metabolic changes. **A** *Pax7*, *MyoD*, and *Nudfs8* expression in *Ndufs8*-overexpressed MBs from TA as quantified. Data are presented as the mean ± SE (*n* = 4). **B** *Ndufs8*-overexpressed MBs were cultured in a growth medium with EdU. The number of EdU(+) cells was counted. Data are presented as the mean ± SE (*n* = 4). Scale bar, 100 µm. **C** and **D** *Ndufs8*-overexpressed MBs were cultured in a differentiation medium for 4 days and stained against EdU (green) and MyHC (red) with DAPI (blue). The proportion of MyHC(+) cells among total nuclei (**C**) and the number of MyHC(−)EdU(−) reserve cells (**D**) were quantified. Data are presented as the mean ± SE (*n* = 4). Scale bar, 100 µm. **E** *Ndufs8*-overexpressed MBs were cultured under normal or serum-starved conditions for 12 h and stained against cleaved caspase-3 (red) with DAPI (blue). The proportion of cleaved caspase-3(+) cells among total nuclei were quantified. Data are presented as the mean ± SE (*n* = 4). Scale bar, 100 µm. **F** *Ndufs8*-overexpressed C2C12 cells were stained with MitoTracker®. Scale bar, 7.5 µm. **G** and **H** OCR in *Ndufs8*-overexpressed MBs was measured after treatment with oligomycin and FCCP (**G**), and basal OCR and SRC of myotubes were quantified (**H**). Data are presented as the mean ± SE (*n* = 3). **I** and **J** ECAR in *Ndufs8*-overexpressed MBs was measured after treatment with glucose and oligomycin (**I**), and basal ECAR and glycolytic capacity of myotubes were quantified (**J**). **K** The expression level of *PGC-1a*, *Nrf1*, and *Tfam* in *Ndufs8*-inhibited MBs was quantified by qPCR. Data are presented as the mean ± SE (*n* = 4).

In investigating the function of *Ndufs8* in MBs, Ndufs8 expression was also overexpressed by a retrovirus vector in TA-derived MBs (Fig. 4A). MyoD expression was not altered, although Pax7 increased in Ndufs8-overexpressed MBs compared with control (Fig. 4A). A proliferation assay showed no difference in the number of EdU(+) cells between control and Ndufs8-expressing MBs (Fig. 4B). These results are contrary to the loss of functions results (Fig. 3). Mitochondrial dysfunction can suppress cell proliferation [28]. However, no direct evidence has been found to explain the mechanism by which mitochondrial function can directly affect *MyoD* expression. A previous study also reported that the overexpression of *Pgc-1α*, a gene related to mitochondrial biosynthesis, had no effect on *MyoD* expression and MB proliferation [29], which is consistent with our observation. In addition, *MyoD* expression is upregulated in proliferating SCs whereas *MyoD*-deficient cells exhibit a high proliferative potential and a strong ability to form SCs [30, 31]. Hence, the change in *MyoD* expression caused by *Ndufs8* expression may be independent of the characteristics of proliferating and self-renewing cells.

Two and four days after induction of MB differentiation, we observed a lower ratio of DAPI(+) cells in MyHC(+) myotubes in total nuclei for Ndufs8-expressing MBs compared with the control group (Fig. 4C). Conversely, the number of MyHC(−)EdU(−) reserve cells significantly increased in Ndufs8-expressing MBs compared with the control group (Fig. 4D). In addition, apoptotic cells under starved conditions were significantly reduced in *Ndufs8*-expressing MBs compared with control (Fig. 4E). These observations indicated that *Ndufs8* is a critical factor maintaining the myogenic properties in MBs.

### Ndufs8 modulates mitochondrial formation and cellular metabolism in MBs

Ndufs are a major accessory subunit of complex I, and the loss of this subunit caused by mutations could lead to mitochondrial complex I deficiency, resulting in mitochondrial diseases, including Leigh syndrome [32, 33]. Therefore, this study attempted to evaluate whether *Ndufs8* expression modulates cellular metabolism in MBs. First, *Ndufs8*-overexpressed TA-derived MBs were stained with MitoTracker® to monitor the shape of the mitochondria in MBs. Control cells exhibited more elongated mitochondria, whereas the fragmented phenotype was observed in *Ndufs8*-overexpressed MBs (Fig. 4F).

Next, the oxygen consumption rate (OCR) and the extracellular medium acidification rate (ECAR) were assessed to evaluate the metabolic effects of *Ndufs8* in MBs, which quantify mitochondrial respiration and glycolysis using an extracellular flux analyzer. Skeletal muscle cells and fibers use oxidative phosphorylation and aerobic glycolysis for energy supply, which are complexity balanced [34]. The OCR in *Ndufs8*-induced MBs was lower than control under basal conditions (Fig. 4G and H). After the addition of carbonyl cyanide-*p*-trifluoromethoxyphenylhydrazone (FCCP), the maximal flux and spare respiratory capacity, defined as the quantitative difference between the maximal and basal OCRs [35], in *Ndufs8*-induced MBs were comparable to control (Fig. 4G and H). However, *Ndufs8*-induced MBs exhibited lower glycolysis as indicated by decreased ECAR in response to the added glucose and lower glycolytic capacity, which is the maximum rate of conversion of glucose to pyruvate or lactate after the addition of oligomycin (Fig. 4I and J). Finally, the expression of the mitochondrial biogenesis markers *PGC-1a*, *Nrf1*, and *Tfam* was also assessed in *Ndufs8*-inhibited MBs. As shown in Fig. 4K, the expression level of these genes was significantly decreased by *Ndufs8* suppression (Fig. 4K). Collectively, these observations indicated that *Ndufs8* expression in MBs strongly affects cellular metabolism.

### Ndufs8 affects p53 acetylation by modulating the intracellular NAD/NADH ratio and Sirt activation

**Fig. 5:**
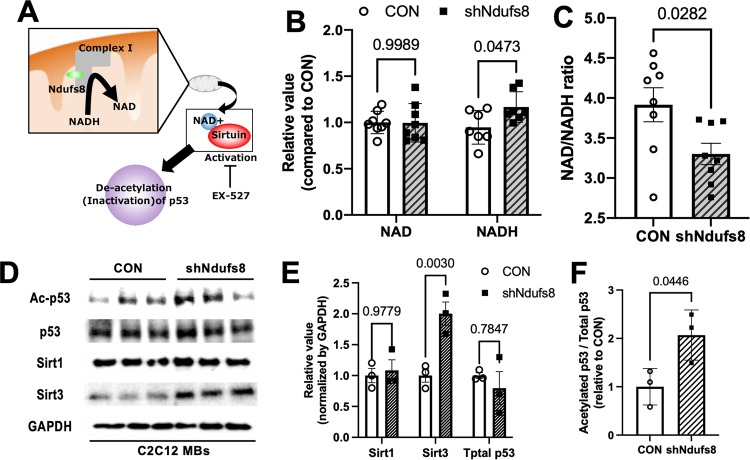
*Ndufs8* affects the intracellular NAD^+^/NADH ratio, resulting in the modulation of p53 acetylation. **A** Schematic image of Sirt-p53 signaling mediated by *Ndufs8*. **B** and **C** Intracellular NAD^+^ and NADH contents (**B**) and NAD^+^/NADH ratio (**C**) were measured. Data are presented as the mean ± SE (*n* = 8). **D**–**F** Protein levels in p53, Sirt1, and Sirt3 during MB proliferation (**D**), indicating that *Ndufs8* knockdown induced the elevation of Sirt3 levels (**E**) and the decline of p53 acetylation ratio (**F**). Data are presented as the mean ± SE (*n* = 3).

Previous reports demonstrated that the deficiency of mitochondrial complex I in mouse models displayed an apparent reduction in the NAD^+^/NADH ratio, resulting in the decline of mitochondrial O_2_ consumption [36–38]. In addition, the suppression of *Ndufs8* expression may affect NAD or NADH accumulation and the altered redox state in the mitochondria of MBs because mitochondrial complex I is the major site for NADH oxidation (Fig. 5A). In evaluating the effects of Ndufs8 on NAD^+^ and NADH levels in MBs, intracellular NAD^+^ and NADH contents were measured, and NADH significantly increased in *Ndufsd8*-inhibited MBs with no change in NAD^+^ levels (Fig. 5B), resulting in the decrease of the NAD^+^/NADH ratio (Fig. 5C).

Sirtuin enzymes, which possess either mono-ADP-ribosyltransferase or deacetylase activity, are critical regulators of mitochondrial and oxidative metabolism by modulating several genes, including *PGC-1a*, *PPARg*, or *p53* [39–41]. Considering that Sirt enzymes are NAD^+^-dependent protein deacetylases, NAD^+^ levels or the NAD/NADH ratio can be rate limiting for the deacetylase activity of Sirt [42, 43]. *Ndufs8*-inhibited MBs showed a significant decline in the NAD^+^/NADH ratio (Fig. 5C), which might reduce Sirt activities. In examining the downstream effects of Sirt activity in MBs, this study measured the protein expression of Sirt1, which is highly expressed in skeletal muscles, mitochondrial Sirt3, and total and acetylated p53 in *Ndufs8*-inhibited MBs. The tumor suppressor protein p53 regulates several genes that lead to cell cycle arrest or apoptosis, and this protein is regulated by Sirt1 [44, 45]. In this study, Sirt3 expression increased, whereas no changes were observed in Sirt1 and total p53 of *Ndufs8*-inhibited MBs (Fig. 5D and E). In addition, a significant increase of acetylated p53 levels in the total p53 protein was observed (Fig. 5F). Given that higher acetylation levels of p53 were observed in cardiac myocytes after apoptosis induction by H_2_O_2_ because of impaired Sirt1 deacetylase activity [46], apoptosis induction observed in *Ndufs8* inhibition could be due to the increase of p53 acetylation caused by the decrease in the NAD^+^/NADH ratio.

In confirming whether p53 acetylation and apoptosis observed in MBs were regulated by Sirt1 activation, MBs were treated with the Sirt1 selective inhibitor Ex-527. Treatment of Ex-527 did not affect Sirt1 and Sirt3 proteins and total p53 levels (Supplementary Fig. S2A and B), whereas the ratio of acetylated p53 levels to total p53 protein significantly increased (Supplementary Fig. S2C), which is consistent with previous results [41]. In the evaluation of myogenic cell properties, Ex-527 treatment decreased EdU(+)-proliferating cells (Supplementary Fig. S2D), but such a treatment did not affect MB differentiation and reserve cell formation in vitro (Supplementary Fig. S2E and F). Moreover, the number of apoptotic cells in Ex-527-treated MBs increased compared with control (Supplementary Fig. S2G). Therefore, p53 acetylation mediated by Sirt1 activation could be a possible factor increasing cell cycle arrest and apoptosis, as observed in *Ndufs8*-inhibited MBs.

### NAD+ supplementation can restore reserve cell formation and protect Ndufs8-knockdown MBs from apoptosis

**Fig. 6:**
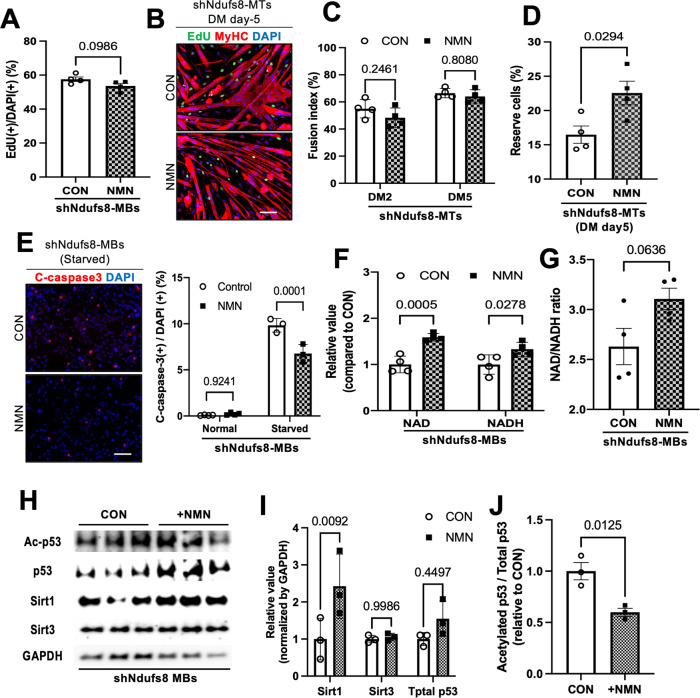
NAD^+^ supplementation (NMN) can ameliorate reserve cell formation and protect *Ndufs8*-suppressed MBs from apoptosis in vitro. **A** *Ndufs8*-suppressed MBs were cultured in a growth medium with NMN and EdU. The number of EdU (+) cells was counted. Data are presented as the mean ± SE (*n* = 4). Scale bar, 100 µm. **B**–**D** *Ndufs8*-suppressed MBs were cultured in a differentiation medium with NMN for 5 days and stained against EdU (green) and MyHC (red) with DAPI (blue; **B**). The proportion of MyHC (+) cells among total nuclei (**C**) and the number of MyHC (−) EdU (−) reserve cells (**D**) were quantified. Data are presented as the mean ± SE (*n* = 4). Scale bar, 100 µm. **E** *Ndufs8*-suppressed MBs were cultured under normal or serum-starved conditions with NMN for 12 h and stained against cleaved caspase-3 (red) with DAPI (blue). The proportion of cleaved caspase-3(+) cells among total nuclei were quantified. Data are presented as the mean ± SE (*n* = 4). Scale bar, 100 µm. **F** and **G** Intracellular NAD and NADH contents (**F**) and NAD/NADH ratio (**G**) were measured in *Ndufs8*-suppressed MBs treated with NMN. Data are presented as the mean ± SE (*n* = 4). **H**–**J** Protein levels in p53, Sirt1, and Sirt3 in *Ndufs8*-suppressed MBs treated with NMN (**H**), indicating that NMN treatment induced the elevation of Sirt1 level (**I**) and the decline of p53 acetylation ratio (**J**). Data are presented as the mean ± SE (*n* = 3).

Considering that the NAD^+^/NADH ratio declined and the myogenic properties were impaired in *Ndufs8*-inhibited MBs, the normalization of the NAD^+^/NADH ratio with nicotinamide mononucleotide (NMN), a NAD precursor that can increase intracellular NAD^+^ levels [47], could improve myogenic cell abilities. NMN treatment did not affect cell proliferation, differentiation, and reserve cell formation in normal MBs, but such a treatment significantly prevented apoptosis under starvation conditions (Supplementary Fig. S3A–D). However, NMN supplementation in *Ndufs8*-inhibited MBs improved reserve cell formation and prevented apoptosis, but it did not affect cell proliferation and differentiation (Fig. 6A–E). Moreover, the number of MyHC(−)EdU(+) cells was higher in control cells than in NMN-treated cells (Supplementary Fig. S3E). We defined reserve cells as MyHC(−)EdU(−) cells, indicating that NMN-treated cells increased the number of EdU(−)MyHC(−) reserve cells. *Ndufs8*-knockdown MBs exhibit a higher percentage of MyHC(−)EdU(+) cells compared with normal cells under differentiation induction conditions (Supplementary Fig. S3F). Therefore, the decrease in *Ndufs8* expression may result in impairments in the transition from proliferating cells to self-renewing ones. Our data indicated that NMN treatment may redirect MBs to arrest their proliferation program and instead transition to a self-renewal pathway under differentiation conditions.

This study also evaluated the NAD^+^/NADH ratio and the protein expression of Sirt1, Sirt3, and p53 and found that NMN treatment increased NAD^+^, NADH content, and NAD^+^/NADH ratio (Fig. 6F and G). In addition, NMN significantly decreased the ratio of the acetylated p53 level to total p53 protein but increased Sirt1 expression (Fig. 6H–J). These observations indicated that the decline of acetylated p53 levels by NMN treatment could improve the impairment of myogenic cell functions observed in *Ndufs8*-inhibited MBs, which would be a possible therapy for aged MBs, where *Ndufs8* levels declined.

### NMN treatment effectively improves SC self-renewal in vivo

**Fig. 7:**
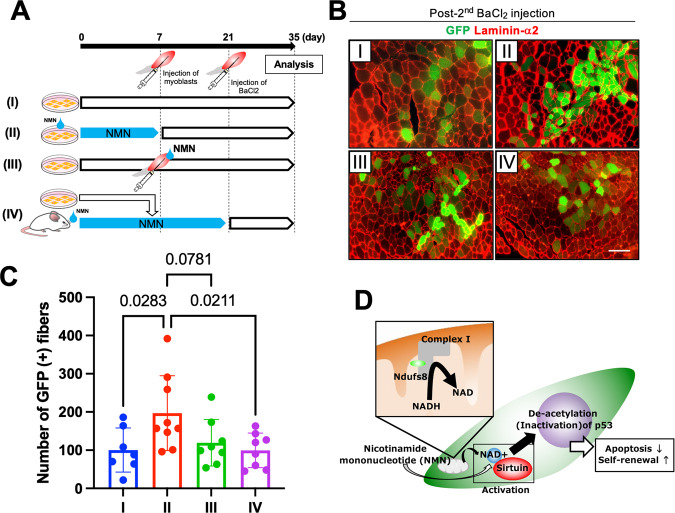
NMN treatment in MBs can improve the efficacy of cell transplantation in vivo. **A** Scheme for MB transplantation experiments. GFP-positive MBs (1.5 × 10^5^ cells) were transplanted into BaCl_2_-injected TA of *NOD/scid* mice. Two weeks after transplantation, engrafted muscles were analyzed or injected with BaCl_2_ to reinduce muscle regeneration. Two weeks after the second BaCl_2_ injection, transplanted muscles were analyzed. GFP (+) donor cells were cultured under growth conditions with NMN (II) or without NMN (I, III, VI). GFP (+) cells were injected simultaneously with NMN solution (10 mM) into TA of *NOD/scid* mice (III) or engrafted into NMN-administered recipient mice (VI). **B** and **C** Two weeks after the second BaCl_2_ injection, cross-sections of transplanted TA were stained against GFP (green) and laminin-α2 (red; **B**), and the number of GFP (+) fibers was quantified (**C**). Data are presented as the mean ± SE (I: *n* = 7, II: *n* = 9, III: *n* = 8, VI: *n* = 8). Scale bar, 100 µm. **D** A model for the regulation of p53 acetylation modulated by *Ndufs8* in myogenic cells.

MB transplantation was performed to confirm the abovementioned observations and assess whether NMN treatment preferentially contributes to SC self-renewal in vivo. Cultured MBs from green fluorescent protein (GFP) transgenic mice (8–12 weeks old) were transplanted into injured TA. Three weeks after transplantation, engrafted TA was reinjured by a second injection of barium chloride (BaCl_2_) to evaluate engrafted MB self-renewal, as described previously [18, 48] (Fig. 7A). Two weeks after the second injury of TA, the number of GFP(+) fibers was counted. In evaluating the effects of NMN in MBs, four different groups were prepared as follows: (I) injection of normal MBs into normal recipient mouse muscles; (II) injection of MBs, cultured with NMN (200 nM) for 5 days, into normal recipient mouse muscles; (III) injection of normal MBs into muscles of recipient mice, where NMN was administered through drinking water (ad libitum in drinking water; 300 mg/kg/day), as described previously [49]; and (VI) injection of normal MBs suspended in NMN solution (10 mM) into normal recipient mouse muscles (Fig. 7A). As shown in Fig. 7B and C, the number of GFP(+) fibers significantly increased in muscles where NMN-treated MBs were injected compared with other muscles. These results indicated that NMN treatment in MBs was effective in improving the self-renewal ability of myogenic cells.

## Discussion

### Muscle SC properties and fiber types

Although the abilities of muscle SCs were thought to be involved in the fiber types where they were derived, this study indicated that SC potentials were maintained even after muscle fiber type transition induced by denervation (Fig. 1C and D). This finding was consistent with previous studies, that is, the muscle fiber type distribution was maintained after injury even in denervated muscles [20, 21], indicating that myogenic functions in SCs could be independent of muscle fiber types. Furthermore, a recent study demonstrated that the regional specific patterns as positional memory in SCs were maintained by Homeobox-A cluster genes [50]. Proteomic analysis of TA-, SOL-, and QF-derived MBs also demonstrated that the protein expression level was completely different, although the distribution of muscle fiber type was similar between TA and QF MBs (Fig. 1F and G). Consistently, previous results using chick embryos with surgical transplantation experiments demonstrated that early limb bud environment defined the proportion of MBs capable of forming slow and fast fiber types and that limb MB diversity arises before the entry of MBs into the limb [51]. These observations supported the hypothesis that myogenic functions in SCs depend not on muscle fiber types but on the muscle region they reside in. The reason why the myogenic functions varied depending on the region of original muscles and the mechanism by which these differences were genetically defined remain unknown. However, as SOL MBs efficiently formed myofiber and SCs after cell transplantation [18], intrinsically understanding the diversity of MBs is necessary to select possible cell sources for cell transplantation therapy. In addition, investigating the diversity in MBs may provide new insights not only into various abilities in MBs but also into the functional differences in myofibers formed by MBs from different regions of muscles.

### Modulation of myogenic cell functions by Ndufs8 and involvement with aging

Based on proteomic analysis, analysis of GO terms downregulated in SOL MBs compared with TA MBs demonstrated links to mitochondrial function (Supplementary Fig. S1A and B). In particular, several protein isoforms related to mitochondrial complex I, including Ndufs4 or Ndufs8, were identified and quantified in SOL MBs. Reportedly, the lack of these genes led to a defect in the mitochondrial structure, resulting in the impairment of cellular metabolism observed in mitochondrial diseases. The suppression or deletion of *Ndufs4* led to the imbalance of the NAD^+^/NADH ratio and metabolic changes, leading to cell death or senescence [37, 38]. Our study also revealed that *Ndufs8* inhibition induced the decrease of myogenic potentials with the decline of apoptosis resistance and metabolic activity (Fig. 3). We could observe an increase in Pax7 expression in MBs when Ndufs8 was overexpressed or suppressed (Figs. 3A and 4A). Pax7 is a crucial transcription factor that plays a key role in regulating the potential of myogenic cells and maintaining SC potential [6]. However, the impact of Pax7 expression on SC self-renewal remains unclear. In a previous study conducted by Rocheteau et al. using Pax7-nGFP reporter mice, Pax7-high myogenic cells exhibited lower metabolic activity and self-renewal potential [52]. Conversely, a study conducted by Lepper et al. demonstrated that quiescent adult SCs do not necessarily require Pax7 for self-renewal and regeneration [53]. In addition, Gayraud-Morel et al. found that Myf5 heterozygous SCs, which displayed enhanced self-renewal capacity compared with WT cells, did not affect Pax7 expression [48]. These findings indicate that Pax7 expression may not be the sole determinant of SC self-renewal. Thus, the defect in the mitochondrial complex I assembly could be a critical component for maintaining living cells.

Previous studies compared SCs obtained from young and aged mice to investigate SC senescence and found that mitochondrial OXPHOS and TCA cycle genes, including *Ndufb5*, consistently decline with age [54], indicating that senescence in SCs may be involved in mitochondrial dysfunction. As shown in Figs. 3 and 4, *Ndufs8* inhibition decreased the potencies in myogenic cells and mitochondrial metabolism, which are comparable phenomena to aged SCs [54]. However, no differences in *Ndufs8* expression in MBs from normal and DMD model mouse muscles were observed, suggesting that this reduction is a specific phenomenon observed in aging cells. In addition, the defects observed in *Ndufs8*-inhibited cells were recovered by the activation of mitochondrial complex I with NMN supplementation (Fig. 6). Thus, the expression or activation of mitochondrial complex I could be crucial to prevent aging-related mitochondrial disfunction in SCs.

Why is mitochondrial function impaired with age? A possible reason is the accumulation of oxidative stress. Oxidative stress, potentially introduced by mitochondrial respiration, decreases mitochondrial DNA content or functions [55]. However, a previous study demonstrated that mitochondrial oxidative respiration is important for the functional maintenance of multiple SCs during aging [54] and that the reduction in cellular NAD^+^ pools weakens the stress-responsive signaling via the mitochondrial unfolded protein response [56], resulting in a loss of mitochondrial homeostasis with the accumulation of reactive oxygen species and a reduction in the number and self-renewal capacity of SCs. This study demonstrated the suppression of basal OCR and ECAR in *Ndufs8*-overexpressed MBs, although these cells had high myogenic potentials (Fig. 4). Thus, *Ndufs8* could reduce oxidative stress with effective ATP synthesis, thereby maintaining mitochondrial homeostasis and protecting SCs from senescence.

### NAD+ is a therapeutic target for aging therapy

In this study, NMN treatment in MBs could increase the efficiency of myofiber formation and SC reconstruction, whereas the administration of NMN into recipient mice could not enhance such processes (Fig. 7B and C). It is considered that the dose or duration of NMN administration was not sufficient to reconstruct the niche, which was suitable for MB survival in recipient muscles. Some studies showed that the short-term administration of NAD^+^ could ameliorate insulin secretion and response in mice with diet- and age-induced diabetes or obesity [47, 57], whereas the chronic administration of NMN could improve age-related tissue dysfunction [49] but could not address the weakness in dystrophic muscles [58]. Therefore, time is necessary to reorganize the deterioration for the improvement of MB transplantation efficacy. In addition, as this study used young mice as recipients, the effects of NMN administration on MB transplantation might have been obscured. If aged mice were used as recipients where the tissue environment would deteriorate, then improvement in MB transplantation efficiency by NAD administration could have been achieved. The deterioration of the muscle environment in aged muscles could reduce MB engraftment efficiency [59], and the improvement of extracellular niches could enhance their efficacy [16]. This point will be explored in the next study.

By contrast, NMN treatment in cultured MBs could improve the self-renewal ability in vitro and in vivo (Figs. 6 and 7), suggesting that NMN changed the essential myogenic function in MBs. Several studies previously reported that myogenic cells from aged mice had an impaired capacity to reconstitute myofibers and replenish stem cells in vivo after transplantation and that these impairments were due to the elevated activity of the p38a and p38b mitogen-activated protein kinase (MAPK) pathways [11, 12] or the upregulation of Stat3 signaling [22]. These age-associated defects in MBs were repaired by treatment with their inhibitors. In addition, SIRT1 activation could protect the heart from oxidative stress via the inhibition of the p38 MAPK pathway [60] or suppress Stat3 signaling during gluconeogenesis [61], indicating that NMN supplementation would regulate these signaling processes. Therefore, as NMN treatment showed protective effects on Ndufs*8*-inhibited cells that possessed similar phenotypes to aged ones, NMN could be a possible therapy for the rejuvenation of senescent MBs.

## Materials and methods

### Animal experiments

All animal procedures were approved by the Experimental Animal Care and Use Committee of the National Institute of Neuroscience of the National Center of Neurology and Psychiatry (NCNP; approval ID 2019012). C57BL/6 mice were purchased from Nihon Crea (Tokyo, Japan), and *NOD/scid* immunodeficient mice were purchased from Charles River (Tokyo, Japan). GFP transgenic mice were kindly provided by Dr. M. Okabe (Osaka University, Osaka, Japan) [62]. Mice with exon 52-deficient X chromosome-linked muscular dystrophy (*mdx52*) were provided by the Riken BioResource Center [63]. All mice were housed and bred in accordance with standard procedures. NMN (Oriental Yeast Co., Tokyo, Japan) was administered in drinking water ad libitum at 300 mg/kg/day [49].

### Denervation model

C57BL/6 mice were randomly divided into denervated and sham operation groups. The left sciatic nerve of mice was surgically excised along nearly the entire length of the thigh through a small incision made in the mid-lateral thigh under general anesthesia. Four weeks after denervation, the mice were euthanized by cervical dislocation under general anesthesia, and the SOL and GAS were dissected for further analysis. The muscles obtained from the right side were used as control [64].

### Isolation of muscle SCs

Mononuclear cells were prepared from C57BL/6 mouse (1.5–24 months old) muscles and GFP transgenic mice (2–3 months old), as described previously [65]. Cells were suspended with 2% fetal bovine serum (FBS; Invitrogen) in Dulbecco’s modified Eagle’s medium (DMEM; Wako) and stained with anti-CD31-FITC or -PE antibody (1:200; clone 390; eBioscience), anti-CD45-FITC or -PE antibody (1:200; clone 30-F11; eBioscience), anti-Sca1-FITC or -PE antibody (1:200; clone D7; eBioscience), anti-integrin α7 antibody (1:200; clone 3C12; MBL International, Woburn, MA, USA), and anti-mouse IgG Alexa Fluor 647 antibody (1:200; Jackson Immuno Research Laboratory). After staining, cells were isolated by flow cytometry equipped with a SONY FACS SH800S (SONY Biotechnology, San Jose, CA, USA). Isolated cells were cultured in a growth medium consisting of DMEM with 20% FBS, 2.5 ng/mL of basic fibroblast growth factor (Invitrogen), 100 U/mL of penicillin, and 100 mg/mL of streptomycin on culture dishes coated with Matrigel (BD Biosciences) at 37 °C. No mycoplasma contamination was detected. The medium was replaced with DMEM supplemented with 5% horse serum (Invitrogen) and penicillin–streptomycin to induce MB differentiation and reserve cell formation. Cultured cells were treated with NMN (200 nM; Sigma) or Ex-527 (10 mM; Selleck).

### RNA extraction and SYBR Green-based qPCR

Total RNAs were extracted from MBs and myotubes using an RNeasy RNA isolation kit (Qiagen). First-strand cDNA was produced using a Go-script Reverse Transcription Kit (Promega) and mixed with GoTaq® qPCR Master Mix (Promega). The specific primers for mRNA expression used for PCR are listed in Supplementary Table S1. mRNA expression levels were quantified on an ABI StepOne™ real-time PCR machine (Applied Biosystems), according to the manufacturer’s instructions, by using the comparative Ct (DDCt) method [66].

### Gene expression and proteomic analyses

Gene expression profiling was performed using the Affymetrix Clariom D (Thermo Fisher Scientific, Waltham, MA) array at Filgen (Nagoya, Japan), and quantitative analysis of proteomes performed by DIA proteomic analysis was performed at Kazusa Genome Technologies (Kisarazu, Japan). Heatmap and PCA were performed using SRplot (https://www.bioinformatics.com.cn/en) [67], and GO analysis was performed using ShinyGO (v0.77) [68].

### Immunohistochemistry, cytochemistry, and mitochondrial analysis

Cultured cells, fibers, and muscle sections were fixed with acetone or 4% formaldehyde solution and blocked with 1% bovine serum albumin (BSA) in phosphate-buffered saline (PBS) containing 5% goat serum. After blocking, they were stained with the following primary antibodies: anti-MyHC (clone MF20; eBioscience), cleaved caspase-3 (clone 5A1E; Cell Signaling Technology, Danvers, MA, USA), antilaminin-a2 (clone 4H8-2; Sigma), and anti-GFP (EMD Millipore, Billerica, MA, USA). After staining, they were incubated with a secondary antibody conjugated with Alexa-488 and -568 (Molecular Probes). After primary and secondary staining, EdU staining was performed using the Click-iT EdU Imaging Kit (Invitrogen), according to the manufacturer’s instructions. Nuclei were stained with 4,6′-diamidino-2-phenylindole (DAPI). Stained cells or sections were analyzed using a BZ-X810 fluorescence microscope (Keyence, Osaka, Japan). For mitochondrial analysis, MBs were incubated for 1 h with the MitoTracker® Deep Red FM (#8778; Cell Signaling), a dye that stains the mitochondria in live cells. Stained cells were analyzed using a confocal laser scanning microscope (SPF5; Leica).

### Measurement of ECAR and OCR

ECAR and OCR were measured as described previously [18]. Cultured MBs were plated on XF24 cell culture plates (Seahorse Bioscience). OCR was measured simultaneously using a Seahorse XF24 Extracellular Flux Analyzer (Seahorse Bioscience), according to the manufacturer’s instructions. Mitochondrial function was assessed using an XF Cell Mito Stress Test Kit (Seahorse Bioscience), according to the manufacturer’s instructions. Oligomycin (6.25 μM), FCCP (1 μM), and rotenone/antimycin A (1 μM) were injected for a Mito Stress Test. The glycolytic function was assessed using an XF Cell Glycolysis Stress Test Kit (Seahorse Bioscience). Glucose (6.25 μM), oligomycin (1 μM), and 2-deoxyglucose (1 μM) were injected to test glycolysis. After analysis, the protein was extracted from MBs in each well using RIPA buffer (Thermo Fisher Scientific). The protein content was measured using a Pierce™ BCA Protein Assay Kit (Thermo Fisher Scientific).

### Western blotting

Proteins from cultured cells were extracted using cell lysis buffer (125 mM Tris-HCl, 15% glycerol, and 2% sodium dodecyl sulfate) supplemented with a Complete Mini protease inhibitor cocktail (Roche, Meylan, France) and PhosStop phosphatase inhibitor (Roche). Proteins (50 µg/lane) were separated by using 4–20% gradient Tris-glycine gels and transferred to a polyvinylidene difluoride membrane (Millipore). After transfer, the membranes were blocked with 5% nonfat milk in Tris-buffered saline containing Tween 20 (TBS-T) for 1 h at room temperature. The membranes were incubated with primary antibodies diluted with 5% BSA (Sigma) in TBS-T overnight at 4 °C. The following antibodies were used for immunoblotting: anti-acetylated p53 (Lys379), anti-total p53, anti-Sirt3, anti-Sirt1 (Cell Signaling Technology), and anti-glyceraldehyde 3-phosphate dehydrogenase (GAPDH; Santa Cruz Biotechnology, Santa Cruz, CA, USA). After serial washes with TBS-T, the membranes were incubated with anti-rabbit or anti-mouse horseradish peroxidase-conjugated secondary antibodies (Cell Signaling Technology) in TBS-T containing 5% milk for 1 h at room temperature. Signals were detected using an ECL Prime Western Blotting Detection Reagent (GE Healthcare, Buckinghamshire, UK) and a ChemiDoc MP imaging system (Bio-Rad, Hercules, CA, USA). The signals obtained from Western blot analysis were quantified using the ImageJ program (National Institutes of Health).

### NAD/NADH assay

NAD^+^ and NADH in MBs were measured using the NAD/NADH-Glo Assay kit (Promega), according to the manufacturer’s instructions. Luminescence values were obtained by using the GloMax® Discover Microplate Reader (Promega).

### MB transplantation and muscle dissection

Twenty-four hours before MB transplantation, 50 μL of BaCl_2_ (1.2%; Sigma) was injected into TA of *NOD/scid* mice to induce muscle regeneration. Cultured MBs were injected into regenerating TA. Two weeks after transplantation, TA were harvested. For reinjury experiments, 2 weeks after MB transplantation, 50 μL of BaCl_2_ was reinjected into TA, and the muscles were harvested 2 weeks after the second BaCl_2_ injection. The dissected muscles were fixed in 4% formaldehyde solution for 30 min, immersed in 10% sucrose/PBS and then in 20% sucrose/PBS, and subsequently frozen in cooled 2-methylbutane (Sigma) with liquid nitrogen. The frozen muscles were sliced into 8–10 µm cross-sections for immunohistochemistry. After staining with GFP and laminin-α2 antibodies, the entire area of the TA was captured, and the number of GFP(+) fibers was manually counted based on captured images, and the identification was blinded to the experimenter.

### Retrovirus production

A retrovirus-based expression plasmid (pMX) was purchased from Cell Biolabs, Inc. *Ndufs8* cDNA (Genscript) was cloned into pMX vectors (Supplementary Fig. S4). The pMX-Ndufs8 and pMX-empty vectors were introduced into PLAT-E retrovirus packaging cells to generate viral particles. Then, the filtered supernatant containing the viral particles was added to the cultured MBs. Furthermore, MBs infected with the pMX-empty vector were used as control.

### RNA silencing

*Ndufs8* and control short-hairpin RNA lentiviral plasmids were purchased from Sigma. Lentiviral vectors, along with packaging plasmids (MDL/RRE, Rev, and VSV-G), were transfected into HEK293T cells using Lipofectamine 3000 (Invitrogen). Three days after transfection, the viral supernatants were collected, mixed with a Lenti-X lentivirus concentrator (Clontech), and incubated overnight at 4 °C. On the following day, the virus was concentrated by centrifugation at 1500 × *g* for 60 min at 4 °C. The concentrated viruses were added to the cultured MBs to suppress *Nudfs8* expression. Seventy-two hours after induction, *Ndufs8*-inhibited MBs were selected using puromycin.

### Statistical analysis

Statistical analysis was performed using GraphPad Prism version 9.1.2 (GraphPad Software, San Diego, CA, USA). All quantitative data were presented as mean ± standard error. A two-tailed *t-*test was used for comparisons between two groups. For multiple comparisons, data were analyzed using one- or two-way analysis of variance, followed by the Dunnett’s or Tukey’s correction, respectively. *P* < 0.05 was considered statistically significant.

### Supplementary information

Supplementary figure legends

original data files

Reproducibility checklist

Supplementary Table 1

## Data Availability

All data needed to evaluate the conclusions in the paper are present in the paper and/or the Supplementary Materials.
