# Supplementary material for: Inherited myogenic abilities in muscle precursor cells defined by the mitochondrial complex I-encoding protein
Source: Cell Death Dis. 2023 Oct 19;14(10):689. doi: 10.1038/s41419-023-06192-2 (PMC10587152; doi:10.1038/s41419-023-06192-2)
Supplement: Supplementary file 1 — Supplementary figure legends [file 41419_2023_6192_MOESM1_ESM.docx]

**Supplemental figure legends**

**Supplementary Figure S1 Proteomic analysis revealed the individual properties of myogenic cells from TA and SOL muscles.** (A and B) GO Biological Component Analysis (A) and GO Molecular Function Analysis (B) were performed for proteins that were highly expressed in SOL MBs compared to TA MBs.

**Supplementary Figure S2 Sirt inhibition increases p53 acetylation and reduces reserve cell formation.** (A–C) Protein levels in total and acetylated p53, Sirt1, and Sirt3 in Ex-527 (10 mM)-treated MBs (A), indicating that Ex-527 elevated the ratio of acetylated p53 to total p53 (C). (D) MBs were cultured in a growth medium with Ex-527 and EdU. The number of EdU(+) cells was counted. Data are the mean ± SE (n = 4). Scale bar, 100 µm. (E–G) MBs were cultured in a differentiation medium with Ex-527 for 4 days and stained against EdU (green) and MyHC (red) with DAPI (blue; E). The proportion of MyHC (+) cells among total nuclei (F) and the number of MyHC (−) EdU (−) reserve cells (G) were quantified. Data are the mean ± SE (n = 4). Scale bar, 100 µm. (H) MBs were cultured under normal or serum-starved conditions with Ex-527 for 12 h and stained against cleaved caspase-3 with DAPI. The proportion of cleaved caspase-3(+) cells among total nuclei were quantified. Data are the mean ± SE (n = 3).

**Supplementary Figure S3 NMN supplementation does not affect myogenic properties but prevents apoptosis in MBs.** (A) MBs were cultured in a growth medium with NMN (200 nM) and EdU. The number of EdU (+) cells was counted. Data are the mean ± SE (n = 4). (B and C) MBs were cultured in a differentiation medium with NMN for 4 days and stained against EdU and MyHC with DAPI. The proportion of MyHC (+) cells among total nuclei (B) and the number of MyHC (−) EdU (−) reserve cells (C) were quantified. Data are the mean ± SE (n = 4–6). (D) MBs were cultured under normal or serum-starved conditions with NMN for 12 h and stained against cleaved caspase-3 with DAPI. The proportion of cleaved caspase-3(+) cells among total nuclei were quantified. Data are the mean ± SE (n = 5). (E and F) The number of MyHC (−) EdU (+) cells in *Ndufs8*-suppressed MBs treated with NMN (E) and in *Ndufs8*-suppressed MBs from SOL muscle (F) were quantified. Data are the mean ± SE (n = 4).

**Supplementary Figure S4 Schematic diagram of pMX-IRES retroviral vector.**

The retrovirus plasmid was constructed by integrating the Ndufs8 gene into the pMX retroviral vector. Key elements include the long terminal repeat (LTR), which facilitates viral integration, *Ψ* RNA packaging signal, the ampicillin resistance gene (Amp^r^) for bacterial selection, the Puromycin Resistance Gene (Puro^r^) for mammalian cell selection, and the internal ribosome entry site (IRES) for efficient translation initiation.
