# Supplementary figures and images for: Inherited myogenic abilities in muscle precursor cells defined by the mitochondrial complex I-encoding protein
Source: Cell Death Dis. 2023 Oct 19;14(10):689. doi: 10.1038/s41419-023-06192-2 (PMC10587152; doi:10.1038/s41419-023-06192-2)

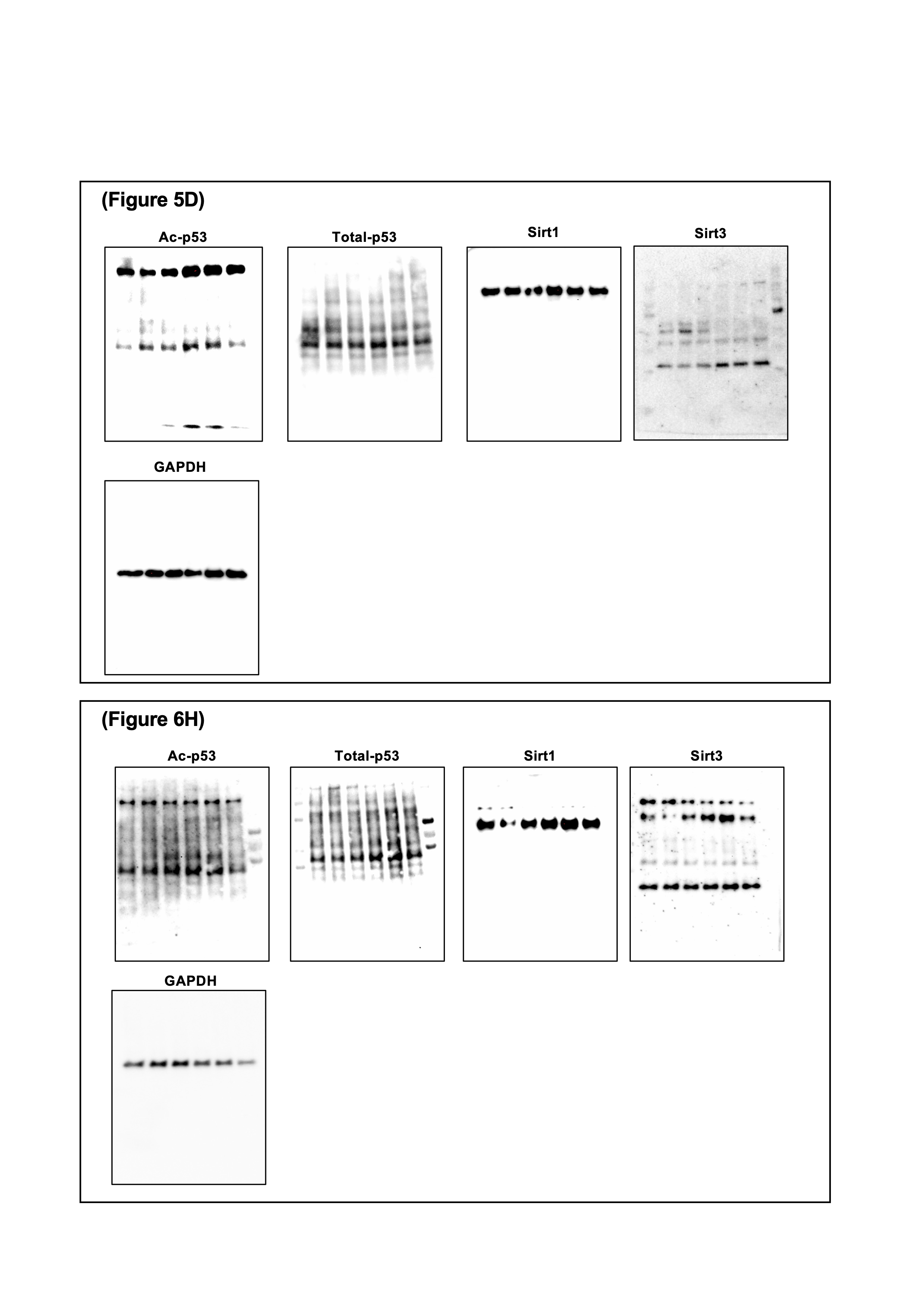

Supplement: Supplementary file 2 — original data files [file 41419_2023_6192_MOESM2_ESM.png]
